# Access to health care for people with stroke in South Africa: a qualitative study of community perspectives

**DOI:** 10.1186/s12913-022-07903-9

**Published:** 2022-04-09

**Authors:** T Smythe, G Inglis-Jassiem, T Conradie, S Kamalakannan, S Fernandes, SM van-Niekerk, R English, J Webster, S Hameed, QA Louw

**Affiliations:** 1 International Centre for Evidence in Disability, London School of Hygiene & Tropical Medicine, London, UK; 2 Division of Physiotherapy, Department of Health and Rehabilitation Sciences, Stellenbosch University, Cape Town, South Africa; 3 Public Health Foundation of India, Indian Institute of Public Health Hyderabad, SACDIR, Hyderabad, India; 4 Department of Global Health and Development, Faculty of Public Health and Policy, London School of Hygiene & Tropical Medicine, London, UK; 5 Department of Physical Therapy & Rehabilitation Science, College of Health Sciences, Qatar University, Doha, Qatar; 6 Division of Health Systems and Public Health, Department of Global Health, Faculty of Medicine and Health Sciences, Stellenbosch University, Cape Town, South Africa; 7 Department of Disease Control, Faculty of Infectious & Tropical Diseases, London School of Tropical Health and Medicine, London, UK

**Keywords:** Stroke, Health service, Access, Equity, Disability, South Africa

## Abstract

**Background:**

Incidence of stroke is increasing in sub-Saharan Africa. People who survive stroke experience disability and require long-term care. Health systems in South Africa (SA) are experiencing important challenges, and services in the public health system for people with stroke (PWS) are fragmented. We aimed to explore the perspectives and experiences of PWS related to stroke care services to inform health system strengthening measures.

**Methods:**

In-depth interviews with 16 PWS in urban and rural areas in the Western and Eastern Cape Provinces of SA were conducted between August and October 2020. PWS were recruited through existing research networks, non-government organisations and organisations of persons with disabilities by snowball sampling. Interviews were transcribed, coded, and thematically analysed. We used the conceptual framework of access to health care as proposed by Levesque et al. to map and inform barriers to accessing health care from the user perspective.

**Results:**

PWS recognised the need for health care when they experienced signs of acute stroke. Health literacy on determinants of stroke was low. Challenges to accessing stroke care include complex pathways to care, physical mobility related to stroke, long travel distances and limited transport options, waiting times and out of pocket expenses. The perceived quality of services was influenced by cultural beliefs, attitudinal barriers, and information challenges. Some PWS experienced excellent care and others particularly poor care. Positive staff attitude, perceived competence and trustworthiness went in hand with many technical and interpersonal deficits, such as long waiting times and poor staff attitude that resulted in poor satisfaction and reportedly poor outcomes for PWS.

**Conclusions:**

Strategic leadership, governance and better resources at multiple levels are required to address the unmet demands and needs for health care of PWS. Stroke care could be strengthened by service providers routinely providing information about prevention and symptoms of stroke, treatment, and services to patients and their social support network. The role of family members in continuity of care could be strengthened by raising awareness of existing resources and referral pathways, and facilitating connections within services.

**Supplementary Information:**

The online version contains supplementary material available at 10.1186/s12913-022-07903-9.

## Background

Stroke is the leading cause of death and disability from non-communicable disease in sub-Saharan Africa [1]. In South Africa (SA), estimates vary between 132,000 to 75,000 people per year having a cerebrovascular accident, and 40,000 stroke-induced deaths per year with one quarter of survivors experiencing disability [2]. Stroke incidence is increasing due to rising prevalence of risk factors such as hypertension, and stroke is also interlinked with the HIV epidemic in the country as the most prevalent neurologic complication of HIV infection [3, 4]. Consequently, young people are also affected by stroke [5] and this places a longer term strain on the health system. People who survive stroke experience disability and require continuum of care at various stages and by various experts including the family during their recovery. This implies a growing need for stroke care in SA, however access to rehabilitation-related services is still lacking or do not reach sufficient quality and capacity [3]. While efforts are made to prevent strokes, provision of stroke care in SA remains largely unmet [6] and where available, is limited in quality and is difficult to access [7, 8]. Resources for rehabilitation and social care for people with stroke (PWS) are also limited in SA [8, 9].

Globally, on average people with disabilities face a range of barriers to accessing care, resulting in worse health outcomes, higher health care costs and poorer service quality [10]. SA has plans to increase universal health coverage (UHC) through provision of national health insurance, however gaps exist in provision of good quality, comprehensive and integrated health services for PWS [7, 8]. The right to health care among people with disabilities is strongly established in SA [11], yet there are gaps between laws, policy and practice.

Whilst there is a growing number of specialised stroke units nationally [2], a mixed-methods study undertaken in 2017 in five public hospitals in the Cape Metro Health District found stroke care varied widely across general medical wards at all hospital levels [12]. Limited access to diagnostic investigations, patient delays in receiving medical attention, and shortages of staff influenced adherence to the SA stroke guideline. The study highlights the need for monitoring systems to enable continuous evaluation of the quality of acute and post-acute stroke services. An observational study of 103 patients at a large provincial hospital found the majority of patients attended their first outpatient rehabilitation appointment (96%; *n* = 77/80), however subsequent low attendance rates were associated with lack of finances, patient migration to other areas, and living a long distance from the hospital [13]. Regarding longer-term stroke recovery, an evaluation study of 30 consecutive patients found that 20 PWS living in a rural community had no access to a rehabilitation facility three months post-stroke. Moreover, they did not get support from government or local authorities, leaving the responsibility to some local non-governmental organisations and families that also had limited resources to provide support [14].

The health systems in SA are therefore experiencing important challenges due to unequal distribution of resources, poor management and leadership crises, increased disease burden, and slow progress in restructuring health care in the democratic era [15]. Nevertheless, improving care for PWS is a priority of the Department of Health.

PWS are a diverse group and learning from communities about their access to health services can inform policy makers on context-appropriate stroke care for the new UHC bill in SA. However, evidence is lacking on which actions are needed to strengthen access to health services for PWS. Our qualitative study aimed to understand access to health care from the perspective and experience of PWS in SA.

## Methods

Our methodological approach and consequent reporting was underpinned by the COnsolidated criteria for REporting Qualitative research (COREQ) statement [16]. Ethics approval for the study was granted from the HREC, Stellenbosch University (Ethics Reference Number N20/05/058) and the London School of Hygiene & Tropical Medicine (LSHTM) (Ethics Reference Number 21883).

### Study design

An exploratory, descriptive, qualitative study within an interpretive research paradigm, using semi-structured individual interviews was undertaken with those experiencing the need for stroke care. The qualitative approach allowed us to explore lived experiences, and gain in-depth understanding of their perceptions, and world views of PWS. In particular, we sought to understand their experiences in accessing health care, their perceptions and preferences with regard to stroke-related health care services, as well as their information and support needs. We then used the codes created deductively within a framework approach to map and inform barriers and enablers for access to stroke services.

**Fig. 1:**
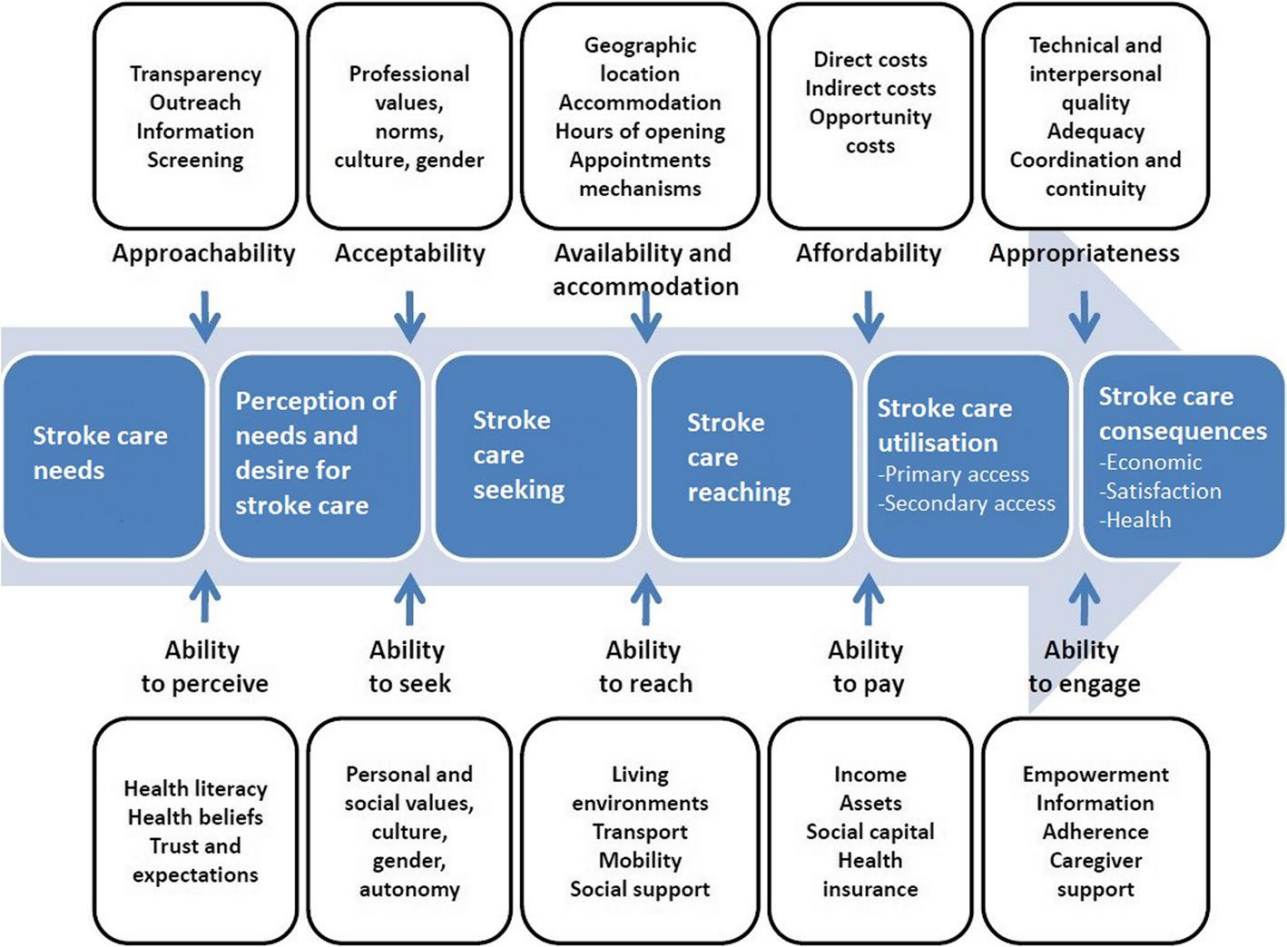
Re-conceptualisation of framework by Levesque et al. [17] for ability to access stroke care

We used the Levesque et al. [17] framework to conceptualise the ability of PWS to access health care. The framework proposes five dimensions of access from both a health systems perspective (supply) and population ability to access (demand). This study sought to explore patient-reported perspectives and experiences related to stroke care services. We therefore used the patient-oriented ‘demand’-side dimensions of the framework. For example, in perceiving the need for health care, the first construct explores the ability of PWS to realise and acknowledge their health care needs as a result of their health literacy, health beliefs, and trust or expectation (Fig. 1).

### Setting

The study was conducted in urban and rural districts of the Western and Eastern Cape Provinces in SA between August to October 2020. The urban district in the Western Cape was the City of Cape Town Metropole and the rural district included the Cape Winelands District. The rural district in the Eastern Cape was the Amathole District and the urban setting was the Buffalo City Metropole.

In South Africa, the National Department of Health develop and endorse policies; health is managed, and policy implementation is governed at provincial level, and implementation occurs at district and sub-district levels. The Western Cape Province consists of urban and semi-urban regions whereas the Eastern Cape Province has many rural and deep-rural areas, which particularly challenges access to health care of marginalised groups such as PWS. The public health sector primarily services the low income and informal settlement populations [18]. The contexts and resource availability within the respective provinces therefore play a major role in the scope and quality of health services [19].

### Sampling and recruitment

Potential participants were recruited from stroke networks known to the research team. These networks included collaborators and/or stakeholders, e.g., stroke support groups and team members previously involved in the South African-Contextualised Stroke Rehabilitation Guideline project and other stroke stakeholder meetings. In addition, one researcher (TC) accompanied by a trained isiXhosa speaking research assistant from the area, recruited participants from the local hospital and surrounding communities in the Mbashe local municipality (in Amathole district) catchment area in the Eastern Cape Province.

The sampling framework included criterion (individuals meeting specified criteria) and snowball sampling through referrals by previously selected participants. Male and female adults above 18 years old who had sustained a stroke within the previous 6 – 12 months were eligible for inclusion. The motivation for this timeframe were: (1) the optimal window period for maximal recovery ranges between the first 3–18 months in the literature and is often also the time when most PWS express the need for stroke care and rehabilitation and support; (2) participants may experience recall challenges if recruited after two years post-incident. Potential participants were excluded when they were unable to understand the aims and processes of the project. All participants were approached through telephone call or email and none refused to participate.

Data were collected at the home of PWS. The majority of participants were interviewed directly. However, carer/proxy interviews were used for people with severe difficulties understanding or communicating with available adaptations (e.g. people who are deaf, illiterate and with no knowledge of sign language; people with severe intellectual/cognitive impairments). Inclusion of PWS was supported through accessible interview sites and transport, and use of researchers skilled at communicating with people with cognitive impairments.

### Data collection

Interview guides with open-ended questions and prompts (Additional file 1) were pilot tested for understanding and administered in English, isiXhosa or Afrikaans by trained research assistants. Questions in the interview guide explored the experience of accessing emergency care for signs of stroke, inpatient and outpatient care, return to home and family support. These draft questions were refined during two online sessions with a panel of researchers and clinicians familiar with stroke care and qualitative research methods, including conducting research with people with disabilities.

The semi-structured interview guide was piloted with a female stroke survivor from an urban setting in the Western Cape province. Based on the pilot interview, the interview schedule was deemed appropriate, clear, and well-phrased and therefore required no changes to the phrasing of the questions. However, the pilot study identified adjustments (pre-interview messages, check interview setting) that would allow a better experience for the participants and these were incorporated into our data collection protocol.

The research assistants (GIJ, TC) are female physiotherapists. Both had qualitative training that included the study protocol and qualitative methods, and a further two-day online and in-person training on data collection, with ongoing mentoring and support provided by the study team (TS and SH). Both interviewers had experience in conducting interviews as clinicians.

The interviewers did not have a prior relationship with any of the participants. Participants were contacted by telephone before the study began and given information about the study, including the reasons for doing the research. Participants were informed about the profession of the interviewer, their affiliation and why the research was being conducted.

### Data management and analysis

All interviews were transcribed *verbatim* and translated to English. Data were managed using AtlasTI (Version 9) and transcripts were labelled for anonymity. Anonymised interview transcripts were analysed using thematic analysis [20]. Initial coding was exploratory and expanded iteratively, identifying key themes emerging from the data. These were discussed across the entire team, and reviewed by experienced researchers (TS, TC, SH and QAL), to ensure that interpretations were credible and valid. For the analysis of access to health care, we applied the framework proposed by Levesque et al*.* [17] to map and inform barriers to access of health care, and disparities in outcomes faced by people with stroke. Frequent discussions with the research team took place throughout the data analysis phase. Participants did not provide feedback on the findings.

### Trustworthiness of the data and processes

#### Credibility

Digital voice recordings were compared with transcriptions for accuracy. Member checking could not be done as many participants did not have access to computers to share the transcripts. We used investigator triangulation and GIJ, TC and SvN made coding and analysis decisions based on a sub-set of the transcripts.

#### Transferability

We provide detailed descriptions of participants and contexts to allow others an opportunity to assess its applicability to their research or context.

#### Dependability

The researchers involved in the interviews kept a record of daily activities concerning research and decisions influencing how the study was carried out.

#### Confirmability

One researcher (GIJ) documented all steps and procedures used for a data audit trail to identify the potential for bias or distortion.

### Ethical considerations

The three main ethical considerations included addressing participant expectations, data protection and sensitive information. We took care to present the nature and detail of our study to avoid raising expectations of participants on the outcome of the interview process. We did not link any data to particular participants. The information sheet describing the study protocols, data management processes, procedures for maintaining confidentiality and plans for data sharing was explained over the telephone and also shared as images on phone messages, and when requested, emailed to a relative to print the forms. The information sheet was reiterated verbally as part of the informed consent process in the language of choice (English, isiXhosa or Afrikaans). The trained research assistants obtained informed consent from all participants (none had significant speech or cognition problems) and carers. The research assistants were trained on identifying need for referral of additional services and services were mapped should referral be required. All interviewees were reimbursed for their time and transport.

## Results

### Description of participants

A total of 16 PWS participated in this study. Demographics of participants are outlined in Table 1.

**Table 1:** Participant demographics

Category		Frequency (n)
Impairment remaining post stroke^a^	Psychological/wellbeing	6
	Intellectual/behavioural	2
	Physical	15
	Speech	2
	Visual	0
Age^b^	18 – 30 years	0
	31 – 40 years	5
	41 – 50 years	3
	51 – 60 years	2
	61 + years	4
Sex	Female	8
	Male	8
Highest education level completed	Primary	5
	Secondary	7
	Vocational training	1
	Nil or unknown	3
Current income source^c^	Pension	5
	Family	3
	Grants	3
	Salary	2
	Nil	2
Setting	Rural	6
	Urban	10

^a^9 participants had more than one impairment ^b^data unknown for 2 participants ^c^data unknown for 1 participant

Data are presented under the five themes that comprise the Levesque et al. [17] framework on health care access: abilities to perceive, seek, reach, pay and engage. Table 2 provides an overview of the themes and sub-themes identified.

**Table 2:** Overview of themes

Themes	Sub-themes
Ability to perceive	Health literacy related to stroke Health Beliefs Spiritual Beliefs Expectations
Ability to seek	Knowledge of health care options, personal values, attitudes and stigma
Ability to reach	Transport options Pathways to care Physical mobility related to stroke Social support
Ability to pay	Out of pocket payments Transport costs Opportunity costs in seeking care
Ability to engage	Health provider-user relationship, inter-personal attributes of health care professionals Caregiver support Satisfaction with care Information/Communication User resilience

#### Ability to perceive: health literacy related to stroke, health beliefs, spiritual beliefs, and expectations

Factors that were found to shape the ability of PWS to realise and acknowledge their health care needs included their health literacy, the mechanism by which they obtained and used health information to make decisions about treatments. The signs of acute stroke such as limb weakness, numbness, blurred vision, dizziness and loss of consciousness, alerted all of the participants and those who assisted them to get medical help:

*When he picked me up, that is when he realised I couldn’t stand and then he called an ambulance. (PWS_female_urban)*

However, in many instances, it was clear that participants and family members had insufficient health literacy to understand the cause of the signs they experienced or underlying factors that may stem from uncontrolled or undiagnosed hypertension.

*The other thing he wasn’t told, was why the one side of his body wasn’t working well at the hospital… (Translator for PWS_male_rural)*

No participant had health literacy on stroke prevention, although some were on medication and engaged with health care services for determinants of stroke.

Health beliefs, such as stroke only affecting those who are older, also contributed to limiting the understanding of what causes stroke and mitigating factors. This could have influenced their ability to perceive risks associated with predisposing conditions, recurrent strokes and prevention. Participants also cited spiritual beliefs about the causes of stroke and reasons for not experiencing a full recovery, which limited their questioning about causes of stroke and how stroke could be prevented.

*It is only God who knows why you have that stroke. If God wants me to have a stroke then who are we to question? (PWS_female_urban)*

A few participants held expectations of medical treatment and explained their unmet expectations of health care professionals and the health system. Participants perceived that their emergency medical situation was not treated as such. They were also given little information about their diagnosis. Suggestions to better meet expectations of PWS included providing a more supportive approach to care:

*I didn’t understand, and I didn’t know what’s happening. I was just so confused. I’d say counselling to prepare you for the future, or maybe just someone to talk to about the experience would help (PWS_female_urban)*

#### Ability to seek (knowledge of health care options, attitudes, and stigma)

The ability of PWS to seek health care related to their knowledge and understanding about health care options and individual rights to obtain the appropriate care. Some participants did not seem to have good understanding of care options. Many participants received physiotherapy and few knew about occupational therapy, mental health and wellbeing support or family support services:

*No, I’m not even sure where you get a counsellor… I don’t know because I didn’t experience or I never had the need to go see a counsellor, so I never bothered with those kind of things. (PWS_female_urban)*

Challenges in seeking care were also experienced with regard long term support. PWS often viewed their need for support as an additional burden on their families and this influenced whether PWS wanted to ask for help. Furthermore, where services may have provided additional support and advice following stroke, the internalisation of negative attitudes (self-stigma) over issues that were perceived to be embarrassing, led to limited support seeking behaviour:

*At first I was very ashamed because I’m so young and I had a stroke and I was so shy to tell people. (PWS_female_urban)*

#### Ability to reach (transport, pathways to care, physical mobility related to stroke, social/family support)

The issues that participants reported in terms of transport were costs of public transport, including taxis (sometimes overcome by sharing), the waiting time for an ambulance and the difficulties of walking. Participants described limited transport options when seeking emergency care for initial signs of stroke. Almost all participants made arrangements with friends or family to cover transport costs, or shared taxi fares to overcome this practical barrier. Participants who managed to get to hospital by an ambulance reported significant delay in the arrival of the ambulance. One participant, who presented to a pharmacy with physical signs of stroke (left sided numbness and weakness), explained her journey to the clinic after the pharmacist was unable to assist with diagnosis:

*I walked because I don’t have a car… and then I... rested three times on the way there and on the way back. (PWS_female_urban)*

None of the PWS interviewed were aware of where to enter the health system; patients can enter the SA health system at any point (for example, they can present to a large tertiary hospital that offers specialise stroke care services without the need for referral or at a primary care clinic that does not offer specialised stroke care). Choice of small clinics and primary care was influenced by the ability of the PWS to move or available transport. Despite delays in diagnosis and treatment, other experiences of timeliness of care were viewed as positive:

*They gave him a good care at [clinic name], they let him to skip the queue to see the nurse first... (Translator for PWS_male_rural)*

Efficient care pathways were limited by the capacity to provide CT scans and medication in the required timeframe since stroke onset. Diagnostic imaging was described as only being available in larger hospitals and the majority of people with signs of stroke had to be transferred to another facility for a scan and diagnosis. They consequently experienced delay in treatment. Although modern treatments such as thrombolysis are available in some central facilities, delays due to transport or efficient emergency care pathways could bar patients from benefitting. One participant was fortunate and noted that he was told that he had arrived within 3–5 h so he could have this treatment to dissolve clots in blood vessels, improve blood flow, and prevent further damage.

Upon return to home, family and social support facilitated participants’ ability to move and mobilise. Family members often provided supportive, caring roles:

*She is coping fine at home. The children are always here and helping her with everything. (Translator for PWS_female_rural)*

PWS described how social support from their families helped them to maintain their care, and contributed to their wellbeing. Families were described as providing physical support, transportation, and finance. Families were also reported as key to reminding participants about rehabilitation exercises to be undertaken at home.

#### Ability to pay (out of pocket payments, transport costs, opportunity costs in seeking care)

The direct costs including insurance and medical aid, indirect costs and opportunity costs of affording the health service all created barriers to access. In the public health sector, services are free if a person is not employed or is a pensioner. However, many of the participants in this study were young. One PWS was referred to a private step-down facility (facility that offers sub-acute and rehabilitation therapeutic care) and reported being unaware of the gap that was not covered by private health insurance:

*They sent me a bill. I don’t even have money. I don’t know how I am going to pay it. It is stressful because they call almost every week. (PWS_female_urban)*

and that some services are not covered by private health insurance at all:

*There was a recommendation that he should also see a psychologist to help with stress, but this is not possible with his medical aid... I feel that the medical aids should definitely add more support in that area. (PWS_male_urban)*

Indirect charges for care, such as paying for transport, and the opportunity costs of rehabilitation, which were driven by length of time travelling, and time not spent tending to family needs were cited as barriers to ability to pay and were seen to be considerable. Additionally, PWS who were primary wage earners found that they could no longer participate in paid work due to their functional impairment and further rehabilitation was limited as finance was required for their basic needs of food and electricity.

#### Ability to engage (health provider-user relationship, caregiver support, satisfaction with care, information/communication, resilience)

There were many reported instances of lack of empathy and negative health provider-user inter-relationships at health facilities, especially related to basic care such as hygiene and mobilising. PWS experienced sudden changes in their functional ability and support of their health journey was perceived to be limited by the engagement of health staff, particularly nursing staff:

*I couldn’t walk to the toilet. I lay there and wet myself. I lay there for a long time – wet. I asked for someone to come help me, and then they walked out of the ward…they are not competent to do that type of work.* (PWS_male_rural)

*My sister had to come every day to see if they had given me the meal correctly and if they changed me. There were nurses just up there...they only came to check every now and then. When you are lying in your urine for a few hours...I don’t want to lie. It wasn’t nice.* (Translator for PWS_male_rural)

The development of a respectful relationship with health care professionals contributed to how the health services were viewed, and the extent to which PWS could engage. PWS particularly valued being treated with kindness by health care professionals and the friendliness of staff made a positive impression on participants. Conversely, negative attitudes hindered engagement of PWS. These personal and social values influenced how PWS were able to engage with their health care.

*They treated me like just another casualty, just like a fool or something (*PWS_female_urban)

*...and the other thing I hate, I hated very much, when I saw him [the doctor] I asked to speak to him in private and he told me specifically that is not an option*…*it’s very personal and it’s very private. It’s my experience and now I must share it with a stranger also.* (PWS_female_urban)

Generally low expectations lead to surprise when treated well and this influenced patient satisfaction and engagement:

*He was happy with the care that he got from the hospital because the nurses were speaking nicely to him, they did not even shout at him. The doctors also spoke nicely to him, no one was shouting at him.* (Translator for PWS_male_rural)

Upon discharge from hospital, although some participants received health information, follow-up communication was sometimes sporadic and resulted in lack of comfort, and limited confidence of caregivers with providing care.

*When he left the hospital, he was told to continue with his medication from the local clinic and do his exercises. He was also told that he will be phoned to come and see physios, but he was never phoned.* (Translator for PWS_male_rural)

Participants experienced inconsistent opportunities to be empowered and engaged in their own health journeys because advice and information about assistive products varied in both rural and urban areas. Participants who were discharged from acute care to home without assistive products used their physical environment for support. Some participants were discharged with more than one piece of equipment, others received wheelchairs of variable sizes and fit, and yet others could not get a walking stick:

She has a wheelchair…and a walking stick (PWS_female_rural)

*...they gave him a small wheelchair, but I thought I had signed for a more comfortable wheelchair* (Carer of PWS_male_rural)

The ability to engage and improve autonomy was also bolstered by printed information. Easy to follow information with graphics was viewed as assisting the participants’ ability to participate in care, and it built trust between the health service provider and PWS. However, in general there was limited opportunity for PWS to be empowered with information, and ability to engage was mainly reported in negative terms:

*I didn’t know what to do and I felt so confused. I went to you as a medical practitioner, who was supposed to guide me but they didn’t. They make you look awful stupid for asking the questions you ask. (*PWS_male_urban)

No participant received support for their children and no services offered family support for children and young families although the need was expressed:

*I think you need to prepare your kids to understand better and not panic when something happens…I’m not that old but still, these things are happening, and they also need to know about it. We need to educate them about these kind of things.* (PWS_female_urban)

The ability to engage and participate in care was enabled by the positive spirit of PWS. Participants and their caregivers demonstrated resilience when seeking and interacting with health care services:

*I also feel a lot stronger and better, and I have a better understanding. I think I’ve accepted what happened, I’ve accepted it because with whatever happened, it happened. I want to get better. I started gardening.* (PWS_female_urban)

We highlight the contextual barriers and enablers to stroke care in the Eastern and Western Cape in Table 3. These experiences and views demonstrate that personal resilience, social and family support facilitate PWS to access stroke care in SA. However, no systems to support and engage the family and carers are in place. PWS had multiple unmet and low expectations of care, giving the impression that PWS were satisfied with the care they received. Improved engagement between health care providers, patients, and carers at critical points along a clear, consistent care pathway will improve care for PWS in SA.

**Table 3:** Contextual barriers and enablers to stroke care in SA

Domain	Barrier	Enabler
Ability to perceive	•Unmet expectations of health care professionals •Low health literacy on stroke prevention •Health beliefs	•Awareness of functional changes requiring medical care
Ability to seek	•Poor understanding of care options •Lack of privacy •Cultural and spiritual beliefs •Stigma	•Being treated with kindness, respecting personal values •Friendliness of staff
Ability to reach	•Limited transport •Complex and variable pathways to care •Physical mobility related to stroke	•Social /family support
Ability to pay	•Out of pocket payments (private health care) •Transport costs •Opportunity costs for seeking care	•Some private health insurance coverage (associated with higher socio-economic status) •Free/scaled costs of public health care
Ability to engage	•Negative health provider – user relationships •Limited engagement of health staff •No support for carers and children of PWS	•Low expectations of good treatment •Printed information and information with graphics •Personal resilience and family support

## Discussion

People with stroke experienced difficulties in accessing health services in rural and urban areas in SA. Our findings demonstrate that PWS recognised the need for health care when they experienced signs of acute stroke, however health literacy on determinants of stroke was low. Challenges to reaching care included complex pathways to care, physical mobility related to stroke, long travel distances and limited transport options. The perceived quality of services was influenced by cultural beliefs, attitudinal barriers and information challenges. The level of adherence and empowerment in the health journey was influenced by information given by health care professionals, provision of assistive products, services that offered family support and personal resilience.

Many of the PWS expressed unmet expectations about the care they received. Acute stroke is regarded as a medical emergency and therefore PWS expected their condition to be treated accordingly. Their expectations included prioritised care provided in a dignified and professional manner. These unmet expectations align with published reports of staff rudeness and low levels of accountability [21–23] with respect to the care of PWS in SA. Negative staff behaviours may be manifestations of low levels of staff competency (e.g., not recognising acute stroke as an emergency), which may be related to the lack of regular upskilling of health care workers in SA on stroke care. Training opportunities for staff could also facilitate better accountability. Staff attitudes are arguably also linked to the complex, intertwined health systems limitations such as workforce shortages and overcrowded facilities [24, 25]. Strategic leadership, governance and better resources at multiple levels are required to address the unmet expectations and health care needs of PWS [26].

PSW recognised the need for care when they experienced signs of acute stroke. However, PWS, their immediate family and community members (neighbours) did not recognise or suspect stroke as the underlying cause of their acute signs. PWS expressed confusion about the cause of their symptoms and desperation to understand their condition and prognosis as they expected to be better prepared for the future. In addition, PWS also appeared to lack knowledge about the underlying risk factors for stroke, despite regular contact with health care facilities for the management of hypertension. These findings are similar to other studies where the greatest proportion of participants who knew of the established stroke signs and symptoms ranged between 18% for paralysis in Uganda and 66% for weakness in Nigeria [6]. Our findings point to the need for information, education, and inclusion of PWS and their families in stroke care, which has been identified worldwide [27]. Many PWS responded positively to information regarding rehabilitation exercises, follow-up appointments and prescribed medication, which indicate a readiness for engaged care. Despite this, our findings show that patient education was not optimal and no information regarding preventative, health promotion and ongoing maintenance for stroke was provided to any of the participants. Since stroke is a chronic condition, ongoing engagement in care is essential [28]. Multiple opportunities exist to educate, activate, and better engage PWS to become an inclusive stroke care system.

PWS in our study reported that personal factors such as privacy and being treated with respect were not always present in the care. Health care workers did not relay personal information privately to patients, further exposing the vulnerability and emotional challenge to deal with sudden onset of physical disability. Although the National Health Act (Act No. 61 of 2003) states that all patients have a right to confidentiality which is consistent with the right to privacy in the South African Constitution, these rights were not always enjoyed by this vulnerable group included in our study. The Protection of Personal Information Act (POPI Act) of South Africa commenced June 2021 and will hopefully serve as a facilitator to revisit the management and communication of personal information within health care settings in SA. Changed health care provider behaviour towards respectful and ethical care for vulnerable groups may contribute to better outcomes and reduced stigma associated with disability.

Participants in our study experienced challenges in accessing transport or delays when trying to get to hospital by ambulance. Our results highlight that and PWS predominantly used primary health care clinics to enter the health system, however these clinics were only located within reasonable distance in urban settings. PWS experienced delayed diagnosis and treatment as transfer to another facility was often required. This underscores the need to provide an effective and efficient emergency service and address the time delay in ambulance arrivals, and raise awareness of routes of access in tertiary hospitals for people experiencing signs of stroke.

Despite numerous challenges to accessibility, throughout the findings there are also indications of new and positive developments. Thus there are mixed findings on access to care in addition to the challenges outlined above, much of which was facilitated by resilience of participants.

### Service implications

Service providers can enhance access to stroke care services by providing information about prevention and symptoms of stroke, treatment, and services to patients and their social support network and providing basic services such as hygiene and mobility assistance for inpatients. Being knowledgeable about existing resources and referral pathways, identifying when PWS need additional support to seek care and facilitating connections within services will strengthen availability and appropriateness of the health system. Stroke services tend to focus on physical impairment, with the entire system managing acute strokes being biomedical [29]. There is a limited psychosocial approach to stroke care, and holistic services to support families, in particular children of people with stroke, are required. Younger people are experiencing stroke in SA and there are no services that support their young dependents. Whilst grants exist to support dependents, access to these requires better engagement between social services and health, and improving referral systems may be required.

### Strengths and limitations

Although our sample was diverse and balanced (variety of ages, equal gender representation), the sample size is relatively small. A larger sample could have improved inter-group comparisons (e.g., differences between age groups, urban and rural dwellers, and educational attainment) and generalisability to different health system contexts. Still, several checks were in place to strengthen the integrity of data and interpretations. These included involving research assistants who collected, translated, and transcribed the interviews in data analysis and interpretation, and ongoing discussions among the whole team throughout data collection and analysis, particularly on our positionality, reflexivity, and how our assumptions as practitioners influenced the research process. Further research should complement the findings of this study by collecting service provider’s perspectives.

## Conclusion

Whilst PWS in urban and rural areas are diagnosed with stroke in SA, there is variability in understanding of causes and the need to seek medical care, as well as expectation for recovery. There was inconsistency and unpredictability in care received by PWS in SA. The level of empowerment of users in the health system for stroke care is low, and PWS experience limited ability to share in informed decision-making. Promising findings relate to the personal characteristics of PWS, such as resilience, the positive attitudes of some health care providers, and awareness of people in both rural and urban areas to attend health services upon onset of stroke signs.

## Supplementary Information

**Additional file 1. **Interview Guide - People with Stroke.

## Data Availability

The datasets generated and analysed during the current study are not publicly available, as the small number of people with stroke makes data potentially identifying, but are available from the corresponding author on reasonable request.

## Abbreviations

EC: Eastern Cape Province
LMIC: Low- and middle-income countries
NGO: Non-government organisation
OPD: Organisation of people with disabilities
PWS: People with stroke
WC: Western Cape Province
