# Supplementary material for: Access to health care for people with stroke in South Africa: a qualitative study of community perspectives
Source: BMC Health Serv Res. 2022 Apr 9;22:464. doi: 10.1186/s12913-022-07903-9 (PMC8993457; doi:10.1186/s12913-022-07903-9)
Supplement: Supplementary file 1 — Additional file 1. Interview Guide - People with Stroke. [file 12913_2022_7903_MOESM1_ESM.docx]

***Appendix 1***

# **Interview Guide - People with Stroke**

### (Focus of interview: To map the patient’s journey detailing experiences of their stroke and perceptions or preferences of Stroke care in SA)

**We want to understand your personal stroke journey, from the start until now. To help us keep track, I would like us to talk about certain main experiences, almost like a road map.**

1. Our first stop will be on the day you had the stroke; Can you tell me what happened that day?
   1. *Back-up Q: Who was with you? How old is your son (OR person that was with them)?*
2. Now, please tell me about getting to a hospital or doctor?
   1. *Back-up Q: How did you get there? Who went with you?*
3. Next, I would like to know what happened when you saw the doctor?
   1. *Back-up Q: Please tell me what the doctor told you.*
4. Sometimes people may stay in hospital for tests, some stay for a few hours while others stay for a few days. Please tell me what happened while you were in hospital?
   1. *Back-up Q: What tests did they do while you were in hospital?*
   2. *Please tell me what the outcome or results of your tests were.*
5. How long were you at the hospital?
6. What happened while you were at the hospital?
   1. *Back-up Q: What kind of therapy did you receive at the hospital?*
7. Next, I would like to know what happened when you left the hospital to go home. What did the staff tell you before you went home?
   1. *Back-up Q: Who was with you?*
8. Now I would like to talk about coming home for the first time, after you left the hospital. Please tell me what happened when you came home.
   1. *Back-up Q: Who was with you when you came home for the first time?*
   2. *Back-up Q: What did you struggle or need help with?*
9. Sometimes people experience changes after a stroke, like changes in walking, talking or dressing. How did your abilities change after the stroke?
   1. *Back-up Q: How did your walking or ability to move around change?*
   2. *Back-up Q: How did your daily tasks, like washing and dressing, change?*
   3. *Back-up Q: How did your thinking or memory, change?*
   4. *Back-up Q: How did your talking or eating change?*
   5. *How has your role in the house changed? (e.g.making food for your family/cleaning and chores/washing clothes, etc).*
10. Sometimes other parts of a person’ life can also change after a stroke. How was your life at home different after the stroke?
    1. *Back-up Q: Please tell me about changes in your family structure.*
11. How was your financial state or money matters different after the stroke?
    1. *Back-up Q: How did the stroke affect your income?*
    2. *Back-up Q: Please tell me about items that you needed to buy after our stroke?*
12. Now, let’s talk about your health in general. How has your health changed after the stroke?
    1. *Back-up Q: How did the stroke affect your health?*
    2. *Back-up Q: Please tell me about the medication that you are taking at the moment.*
    3. *Back-up Q: How did the stroke affect your mood or feelings?*
    4. *Back-up Q: How did the stroke affect your lifestyle and habits (from before the stroke)?*
13. Now thinking of your overall experience with stroke and the treatment you have had so far, please share any suggestions that could make health care better for people with stroke.
14. Now, thinking of one of your neighbours…If they had a stroke, WHAT would you like them to know about stroke?
15. Now, thinking of one of your neighbours…If they had a stroke, WHAT advice would you give them about recovery after stroke?

## Wrap up

- Before we wrap up, is there anything else which I haven’t asked you which you want to share with me?

## Closure of Interview

- Thank you for your time and for sharing your experiences with us.
- Now that we have finished with the interview, I would like to tell you about the Heart and Stroke Foundation of South Africa. They have a website with useful information on stroke and advice on how to cope after it. There are also tips on how to prevent another stroke from happening. <http://www.heartfoundation.co.za/>
- You can phone their head office on 021 422 1586 to get lifestyle advice or assistance to find support services. (email: [heart@heartfoundation.co.za](mailto:heart@heartfoundation.co.za))
- I would like to share the contact details with you. Could I send the details to your phone number or email?
- Enjoy the rest of your day.
- I will stop the recording now (NB! wait until it has stopped before greeting)
- Good bye (Disconnect the call).
